# Multiple fresh fecal microbiota transplants induces and maintains clinical remission in Crohn’s disease complicated with inflammatory mass

**DOI:** 10.1038/s41598-017-04984-z

**Published:** 2017-07-06

**Authors:** Zhi He, Pan Li, Jianguo Zhu, Bota Cui, Lijuan Xu, Jie Xiang, Ting Zhang, Chuyan Long, Guangming Huang, Guozhong Ji, Yongzhan Nie, Kaichun Wu, Daiming Fan, Faming Zhang

**Affiliations:** 1 Medical Center for Digestive Diseases, the Second Affiliated Hospital of Nanjing Medical University, 121 Jiang Jia Yuan, Nanjing, 210011 China; 2 Key Lab of Holistic Integrative Enterology, Nanjing Medical University, 121 Jiang Jia Yuan, Nanjing, 210011 China; 3 Department of Radiology, the Second Affiliated Hospital of Nanjing Medical University, Nanjing, 210011 China; 4 State Key Laboratory of Cancer Biology & Xijing Hospital of Digestive Diseases, the Fourth Military Medical University, Xi’an, 710032 China

## Abstract

The ancient Chinese medical literature, as well as our prior clinical experience, suggests that fecal microbiota transplantation (FMT) could treat the inflammatory mass. We aimed to evaluate the efficacy and safety of multiple fresh FMTs for Crohn’s disease (CD) complicated with intraabdominal inflammatory mass. The "one-hour FMT protocol" was followed in all patients. Twenty-five patients were diagnosed with CD and related inflammatory mass by CT or MRI. All patients received the initial FMT followed by repeated FMTs every 3 months. The primary endpoint was clinical response (improvement and remission) and sustained clinical remission at 12 months. Secondary endpoints were improvement in size of phegmon/abscess based upon cross-sectional imaging and safety of FMT. 68.0% (17/25) and 52.0% (13/25) of patients achieved clinical response and clinical remission at 3 months post the initial FMT, respectively. The proportion of patients at 6 months, 12 months and 18 months achieving sustained clinical remission with sequential FMTs was 48.0% (12/25), 32.0% (8/25) and 22.7% (5/22), respectively. 9.5% (2/21) of patients achieved radiological healing and 71.4% (15/21) achieved radiological improvement. No severe adverse events related to FMT were observed. This pragmatic study suggested that sequential fresh FMTs might be a promising, safe and effective therapy to induce and maintain clinical remission in CD with intraabdominal inflammatory mass.

## Introduction

Crohn’s disease (CD) is characterized by a transmural inflammatory process, which may lead to the formation of intraabdominal inflammatory masses (phlegmon or abscess)^1, 2^. Phlegmons originate from deep fissuring ulcerations, and typically extends to the adjacent mesentery and the affected loop of bowel. Some may further undergo liquefaction necrosis, thus evolving into an abscess. Small inflammatory masses can be treated with antibiotics, with or without percutaneous drainage, whereas the surgical resection of the inflammatory mass is inevitable for refractory cases^3, 4^. However, given concerns regarding postoperative recurrence of Crohn’s disease and short bowel syndrome resulting from multiple surgical operations, surgical treatment is considered as a last resort. Importantly, another particular dilemma for clinicians is whether to continue immunosuppressive therapies for severely active patients in the setting of an inflammatory mass because of the increased risk of abdominal infection^1–4^. Thus, the management of refractory CD complicated with intraabdominal inflammatory mass is challenging.

Fecal microbiota transplantation (FMT), a concept originated from China a millennia ago^5^, shows promise as a treatment for inflammatory bowel disease (IBD) from recent reports^6–9^. In fact, the classic traditional Chinese medicine book “Ben Cao Gang Mu”^10^ clearly recorded that fresh human fecal solution or fermented fecal solution was used for treating indications of *Wenbing* with super-high fever, poisoning, food poisoning, and abscesses. Furthermore, we described the first case of refractory fistulizing CD complicated with a large inflammatory mass successfully treated with FMT^11^. This important case indicated that the inflammatory mass with infection in abdominal cavity might be not the contraindications to FMT. Based on these reports and our preliminary findings from the trials of FMT treating refractory IBD^12–14^, we hypothesized that FMT could induce clinical remission and that subsequent repeated FMTs could maintain that remission with CD related inflammatory mass. Therefore, this pragmatic study was designed to evaluate the safety and efficacy of multiple fresh FMTs for CD complicated with intraabdominal inflammatory mass.

## Results

### Patient characteristics

A total of 145 patients with CD were evaluated. 120 patients (82.8%) were excluded from analysis, including 114 patients not meeting the inclusion criteria on MRI or CT scan, one for the uncertain clinical response to anti-TNF therapy three months before FMT and five patients with less than 12 months follow-up. Finally, 25 CD patients complicated with intraabdominal inflammatory mass were analyzed in this study (Table 1).

**Table1:** The characteristics of included patients.

Items	Results
Total number, n	25
Age, m ± SD (years, range)	36.4 ± 12.63 (18–61)
Sex, male % (n)	52% (13)
Disease duration (years, m ± SD)	6.2 ± 3.91
Harvey Bradshaw Index (m ± SD)	11.0 ± 2.68
Disease location, % (n)	
Ileum	12% (3)
Ileum and colon	76% (19)
Colon	12% (3)
Perianal lesions, % (n)	72% (18)
Steroid dependent before FMT, % (n)	40% (10)
Failed or cannot afford biologic therapy, % (n)	56% (14)
Treatment history, % (n)	
Steroids	68% (17)
Immunosuppressants	60% (15)
Anti-TNFαantibody	28% (7)
Surgery	72% (18)
Diagnosis of inflammatory mass, % (n)	
Phlegmon	88% (22)
Abscess	12% (3)
Inflammatory mass location, % (n)	
Ileum/right lower quadrant	76% (19)
Ascending colon	8% (2)
Descending colon	4% (1)
Sigmoid	8% (2)
Presacral region	4% (1)
Associated fistula,% (n)	52% (13)
Associated stenosis,% (n)	32% (8)

### Clinical response to multiple fresh FMTs

**Figure 1:**
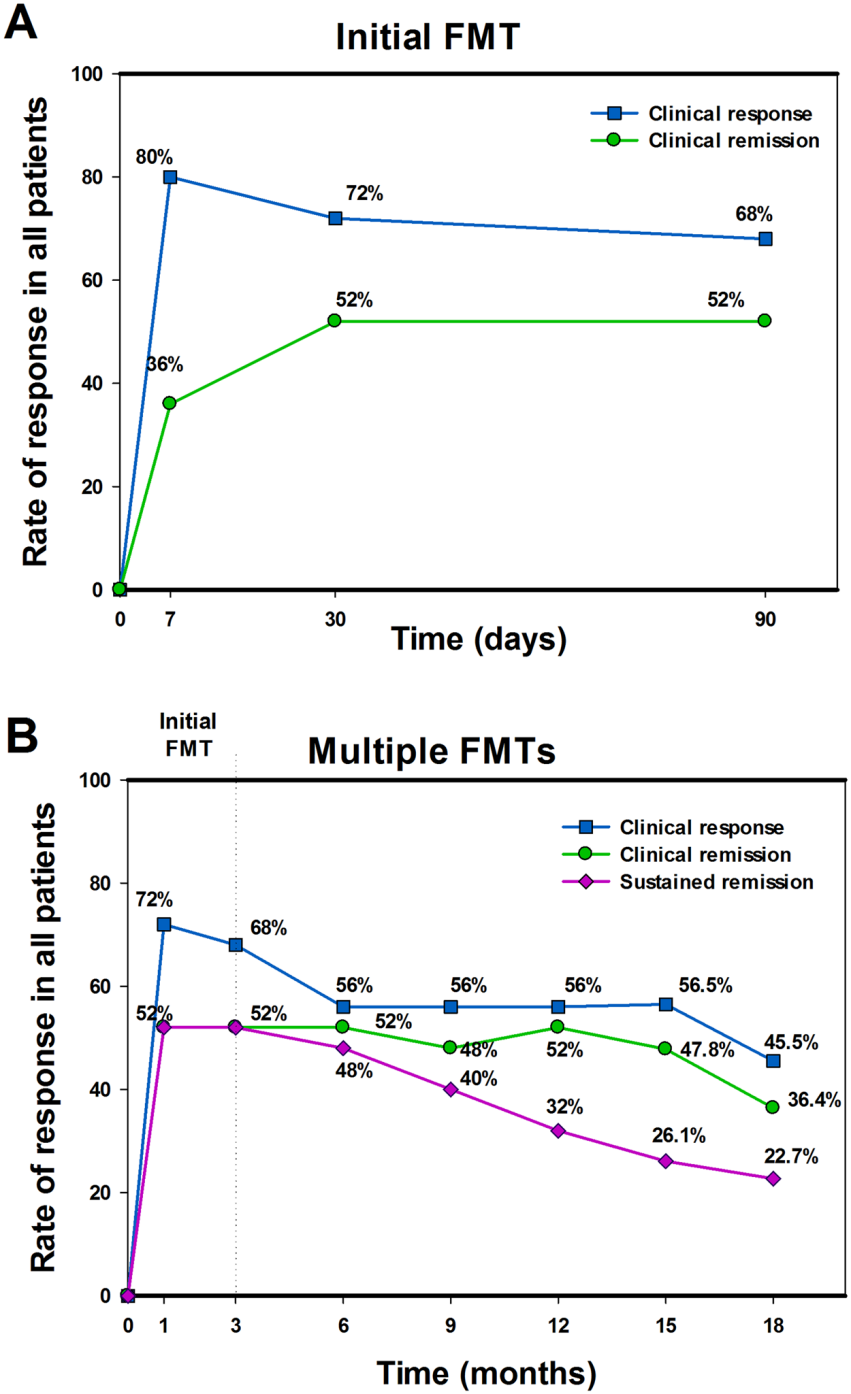
The percentage of FMTs induced response at each assessment point. (**A**) The changing rate of clinical response induced by the initial FMT within three months. (**B**) The changing rate of “response”, “remission” and “sustained clinical remission” during the follow-up at each assessment point. The response included clinical remission and clinical improvement. The remission included patients with sustained clinical remission and patients with relapse/flare but induced to remission by FMT. The sustained clinical remission included patients without any relapse/flare during the follow-up.

Before the initial FMT, seven of total 25 patients received antibiotics (3 to 7 days), including metronidazole by oral or/and enema, or a combination with levofloxacin by intravenous infusion. The rate of clinical response (including improvement and remission), “clinical remission” and “sustained clinical remission” at each assessment point were respectively shown in Fig. 1. 52.0% (13/25) of patients achieved sustained clinical remission at 3 months post the initial FMT. Of the 25 patients, seven chose a longer time to undergo repeated FMTs because of their satisfactory condition and/or inconvenience of traveling to our hospital. With the repeat FMTs, the proportion of patients at 6 months, 12 months and 18 months achieving sustained clinical remission was 48.0% (12/25), 32.0% (8/25) and 22.7% (5/22), respectively. Nine patients experienced relapse during the follow up, but they achieved clinical remission or improvement again with subsequent FMTs. Two patients had an immediate response to the initial FMT but suffered from obstructive symptoms and aggravated abdominal pain within one month and then were successfully treated with gastrointestinal decompression and antibiotics. One patient achieved clinical remission but had a relapse after 15 months, then was induced remission again by the repeat FMT. However, his condition suddenly deteriorated three months after his last FMT with confirmed presence of spontaneous perforation (not related to FMT). Two patients experienced a transient improvement within three months after the initial FMT and then chose switch therapy. One switched to thalidomide due to the limited efficacy of FMT and other therapies (such as, 6-mercaptopurine) leading to leucopenia, another switched to the infliximab due to the uncontrolled perianal fistula discharge. Two patients had no response to either the initial or subsequent FMT, though they experienced partial decrease in symptom severity. Three patients (12%) underwent surgery. One of the three switched to surgery at three months after the initial FMT because of the development of an enterovesical fistula. The second one underwent a stoma creation for keeping refractory intestinal obstruction within one month after the initial FMT. The third benefited from the serial FMTs controlling diarrhea for one year but finally decided to undergo stoma for perianal fistula symptoms.

**Figure 2:**
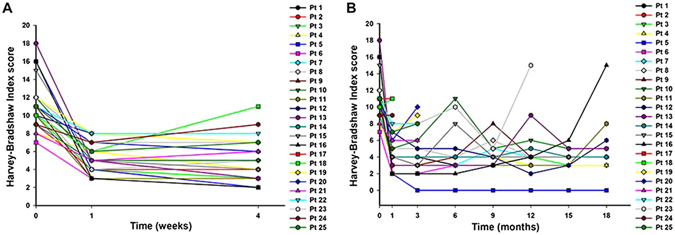
Harvey-Bradshaw Index (HBI) scores over the study period. (**A**) The HBI score at baseline and one month after the initial FMT (n = 25). Nine patients rapidly achieved clinical remission within one week, and 13 had clinical remission within one month. (**B**) The change in HBI scores during follow-up (1–18 months) with multiple FMTs (n = 25). 9 patients experienced relapse or flare with the activity of their diseases were controlled by repeated FMTs. 5 patients eventually achieved sustained clinical remission at the assessment point of 18 months.

The comparison of HBI score between each assessment point from one week to 18 months and baseline was significant (P < 0.05). As show in Fig. 2A, HBI score dramatically decreased in one week after the initial FMT. CRP significantly decreased at the third day after FMT (media 18.50 IQR 11.25-55.25 vs. media 11.0 IQR 9.0–20.0, P = 0.012), while the difference of ESR between before and after the initial FMT was not statistically different (P > 0.05). The change of HBI score at each assessment point showed the maintenance of the benefit from the repeat FMTs (Fig. 2B). Both hemoglobin at three months assessment point (99.18 ± 18.87 vs. 110.27 ± 23.28, p = 0.045) and albumin (33.27 ± 7.77 vs.38.26 ± 8.55, p = 0.036) significantly increased respectively, compared with those before the initial FMT.

### Imaging evaluation

**Figure 3:**
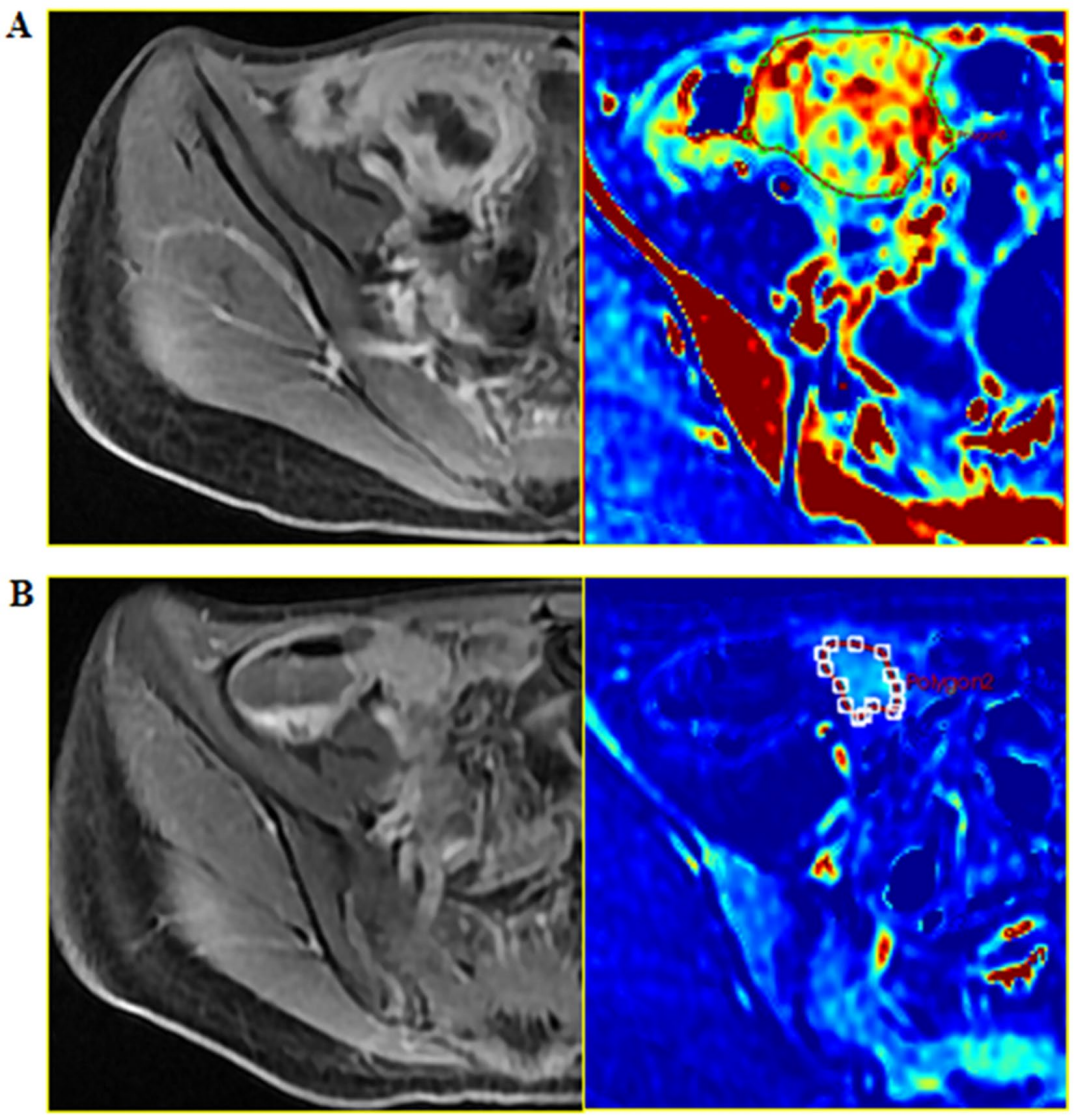
Dynamic contrast-enhanced MRI and diffusion-weighted MRI imaging improved the differentiation of CD and the related inflammatory mass. This case (patient 14) was diagnosed as a fistulizing mass at the terminal ileum ((**A**), before FMT) and was assessed as radiological improvement ((**B**), three months after FMT).

The sizes evaluation of inflammatory mass was shown in the Table 2. The final image data were collected from the first scan and the last scan. Twenty-one of the 25 patients received the image review, 9.5% (2/21) patients achieved radiological healing, 71.4% (15/21) patients achieved radiological improvement, 9.5% (2/21) patients achieved no radiological change and 9.5% (2/21) patients got radiological worsening. Figure 3 showed the image change of patient 14 who achieved obvious radiological improvement.

**Table 2:** Inflammatory mass change and evaluation. RLQ, right lower quadrant; ND, not detected; ATB, antibiotics; FU, follow-up; The size of inflammatory mass was the maximum diameter times the vertical short diameter in the largest cross section.

Case	Diagnosis	CT/MRI	Location	Associate with	Used ATB before FMT	Baseline size (cm*cm)	Size at best time (cm*cm)		Final outcome	
							Within the sixth months of FU	After the sixth months of FU	radiological	Clinical
1	Phlegmon	MRI	Ileum/RLQ	Fistula + Stricture	Yes	5.3*3.6	3.5*2.7	4.4*3.3	Improved	Sustained remission by FMTs
2	Phlegmon	MRI	Ileum/RLQ	None	No	4.8*2.8	3.8*2.9	3.5*2.3	Improved	Sustained remission by FMTs
3	Phlegmon	MRI	Ascending colon	None	No	4.7*3.9	ND	3.9*2.8	Improved	Sustained remission by FMTs
4	Phlegmon	MRI	Ileum/RLQ	Fistula	No	8.0*5.4	ND	7.4*4.3	Improved	Sustained remission by FMTs
5	Phlegmon	CT	Ileum/RLQ	Fistula	Yes	14.0*8.0	3.8*3.3	0.0*0.0	Healed	Sustained remission by FMTs
6	Phlegmon	MRI	Ileum/RLQ	None	No	4.9*3.1	ND	3.9*2.8	Improved	Sustained remission by FMTs
7	Phlegmon	MRI	Ileum/RLQ	Stricture	No	7.9*2.0	7.4*3.5	ND	Worse	Flare or relapse controlled by FMT
8	Phlegmon	MRI	Ileum/RLQ	None	Yes	8.4*3.9	4.7*2.8	5.2*2.3	Improved	Flare or relapse controlled by FMT
9	Phlegmon	MRI	Ileum/RLQ	Fistula	No	8.9*6.2	8.2*5.5	ND	Improved	Flare or relapse controlled by FMT
10	Abscess	MRI	Ileum/RLQ	None	No	9.5*7.3	6.3*5.0	ND	Improved	Flare or relapse controlled by FMT
11	Phlegmon	MRI	Descending colon	Fistula + Stricture	No	5.7*7.7	ND	8.4*5.9	Worse	Flare or relapse controlled by FMT
12	Abscess	MRI	Presacral region	Fistula	Yes	6.1*3.5	5.8*2.7	5.5*2.8	Improved	Flare or relapse controlled by FMT
13	Phlegmon	MRI	Ileum/RLQ	None	No	8.3*5.4	ND	4.8*5.2	Improved	Flare or relapse controlled by FMT
14	Phlegmon	MRI	Ileum/RLQ	Fistula	No	6.9*4.2	3.4*2.9	0.0*0.0	Healed	Flare or relapse controlled by FMT
15	Phlegmon	MRI	Ileum/RLQ	Stricture	No	5.3*5.1	ND	ND	ND	Flare or relapse controlled by FMT
16	Phlegmon	MRI	Ileum/RLQ	None	No	6.1*5.3	5.7*5.4	6.6*5.2	Unchanged	Stable for 18 months but then worse
17	Phlegmon	MRI	Ileum/RLQ	Fistula + Stricture	Yes	7.0*6.1	5.6*5.6	ND	Improved	No response to FMT
18	Phlegmon	MRI	Ileum/RLQ	None	No	6.6*4.8	ND	ND	ND	No response to FMT
19	Phlegmon	MRI	Sigmoid	Fistula + Stricture	No	5.8*5.4	6.3*4.3	ND	Improved	No response to FMT
20	Phlegmon	MRI	Sigmoid	Fistula	No	6.9*5.7	ND	ND	ND	No response to FMT
21	Phlegmon	MRI	Ileum/RLQ	None	No	8.9*8.1	9.5*7.2	ND	Unchanged	No response to FMT
22	Phlegmon	MRI	Ascending colon	Fistula + Stricture	No	8.7*7.9	7.7*6.4	ND	Improved	No response to FMT
23	Phlegmon	MRI	Ileum/RLQ	None	Yes	10.1*6.7	ND	9.0*5.4	Improved	Partial response, switch to stoma
24	Phlegmon	CT	Ileum/RLQ	Fistula + Stricture	No	9.7*7.6	8.4*7.5	ND	Improved	Partial response, switch to stoma
25	Abscess	MRI	Ileum/RLQ	Fistula	Yes	12.4*7.9	ND	ND	ND	Partial response, switch to stoma

### Safety of FMT

There were no severe adverse events related to FMT during the short term after FMT and long term follow-up. Two patients were observed with fever after FMT. One had fever (38.5 °C) and that spontaneously subsided within 6 h after FMT. Another had fever (39 °C) within three days after FMT and was treated with intravenous hydrocortisone, following negative blood cultures, a normal white blood cell and increasing ESR. Three patients had a self-limiting diarrhea within 24 h after FMT. And one patient with perianal fistula developed transient anal pain after FMT that resolved within one day without medical intervention. One patient described above occurred spontaneous perforation at 18 months after the initial FMT and had three months of clinical remission after the last FMT. This might be not related to FMT.

## Discussion

The treatment of intraabdominal inflammatory masses associated with CD is a clinical challenge. While there is some evidence that high dose steroids or anti-TNF therapy can be safe and effective for CD complicated with inflammatory mass if used with proper antibiotic coverage, these medical therapies may not prevent the need for surgery eventually. Felder *et al*.^15^ reported that 58.3% (14/24) of CD patients with a palpable abdominal mass treated with high dose steroids subsequently required surgery. Cullen *et al*.^2^ reported that 15.4% (2/13) of CD patients with abdominal phlegmons eventually had surgery more than a year after starting anti-TNF therapy. Besides, the risk of disease recurrence after surgery is high in penetrating CD, and repeat surgical resection can lead to the short bowel syndrome. Results from this study suggest that the fresh FMT may help to avoid surgery for CD patient with an intraabdominal inflammatory mass.

Evidence suggests that the direct penetration or the remote seeding of bacteria from diseased bowel and transmural inflammation contribute to the formation of inflammatory masses of CD^3^. Keighley *et al*.^16^ reported *Escherichia coli*, *Bacteroides fragilis*, *Enterococci* and *Streptococci* were the most frequently isolated organisms in CD patients with intra-abdominal abscesses. Cartun *et al*.^17^ identified both *E. coli and* various *Streptococci* adjacent to intestinal ulcers and fistula. These results indicated that gut microbiota may be a therapeutic target for treatment of CD-associated inflammatory masses.

In this study, the initial FMT induced clinical remission in 52.0% patients and clinical improvement in 68% patients at three months. The repeat FMTs played a therapeutic role in maintaining clinical remission. With the continued FMT every 3 months, 47.8% to 52.0% patients had clinical remission and 56.0% to 68.0% patients had clinical improvement respectively during the follow up (3–15 months). These results support that fresh FMT can induce remission, and multiple fresh FMTs might be a potential treatment strategy to maintain long-term remission. Similarly, our earlier findings suggest that step-up FMT strategy may offer steroid-sparing benefits for steroid-dependent IBD^14^. In the present study, 60% (6/10) of steroid dependent patients achieved steroid-free clinical improvement, including 20% (2/10) who achieved steroid-free clinical remission. Further study about the gut microbiota affecting the response to corticosteroids is warranted.

Previously reported clinical response rates of FMT for CD have been variable. Vaughn *et al*.^18^ reported single FMT induced clinical response in 58% of patients. Suskind *et al*.^19^ reported that 77.8% of patients with CD achieved clinical remission two weeks after FMT, and our previous reports^12^ showed clinical remission rate of 76.7% at 1 month. In the presently selected population of CD patients with inflammatory mass, the remission rate was 58.8% at 1 month. These variable results may be associated with different conditions, different lab protocols, different donors, different volume of microbiota in suspension and so on. Although FMT with frozen and fresh fecal microbiota are similar in efficacy for treating recurrent *Clostridium difficile* Infection (CDI)^20^, this may not be the case when using FMT for CD. For example, our previous pilot study^12^ indicated that the frozen fecal microbiota had lower clinical response of FMT than the fresh status for CD at 3 months after FMT (42.9% vs. 91.3%, p = 0.016). Notably, all patients in the present study received the fresh rather than the frozen microbiota in their FMT.

Following the first FMT induced remission, the timing of when to give the repeat FMTs remains unclear. Based on our observation, repeat FMT for maintaining the previous FMT efficacy should be within 3–6 months. Our ongoing research will provide stronger evidence in the near future. According to our recent survey, 74.29% patients with IBD were willing to undergo the second FMT^21^. Although we recommended repeat FMT every 3 months, some patients refused due to satisfactory condition and/or inconvenience of traveling to our hospital. These factors might partially contribute to the decreased rate of clinical remission and clinical improvement at 18 months. Some amazing findings were recorded in our study, though there were not shown or analyzed in this article. For example, we did not describe the patient with abscess at 9.5 × 7.3 cm in detail in this article, because we had reported more serious case with inflammatory mass in 2013^11^.

In this study, no severe adverse events related to FMT based on our strict lab protocol and clinical flow (﻿called as the “﻿﻿one-hour FMT ptotocol”) was observed. This suggests that the presence of an inflammatory mass in CD is not a contraindication to FMT. As the disruption of microbial homeostasis is considered as a risk factor for infection^22, 23^, reconstruction of gut microbiome composition by FMT could play protective effect.

There are several limitations to this study. It is a pilot single center trial without control groups. The number of subjects was small, and there was no endoscopic evaluation for each patient. A multicenter randomized clinical trial with a larger sample size is necessary to provide more evidence. Furthermore, the microbial and metabolic analyses for understanding the mechanism were not conducted.

In conclusion, this is the first study with the largest sample of patients to demonstrate that fresh FMT should be a safe and effective therapy for CD complicated with intraabdominal inflammatory mass. Scheduled repeated fresh FMTs may be used to maintain the efficacy of the previous FMT in CD with inflammatory mass. This study opens a new window to the future treatment of CD- related chronic penetrating complication.

## Methods

### Patients recruitment

Patients aged 18–70 years with moderate to severe CD, as defined by Harvey-Bradshaw Index (HBI), were enrolled in this study. Eligible patients were classified as refractory CD according to the criteria in our previous article^12^. The intraabdominal inflammatory mass (including phlegmon and abscess) of the patients was identified by contrast enhanced MRI or contrast enhanced CT before FMT. Concurrent CD medications were held at stable doses for at least one month before screening. Exclusion criteria included: (1) patients accompanied with serious diseases, including other intestinal diseases (e. g. C. difficile infection, cancers, organ failure, heart diseases); (2) patients with refractory obstruction symptoms after conservative treatment; (3) patients who received biological therapies (such as, infliximab) had uncertain clinical response three months before FMT.

### Donor selection

Healthy stool donors aged 10–25 years old were screened by our protocol previously published^12–14^. Briefly, donors did not use antibiotics, laxative or diet pills in the past three months and had no recent gastrointestinal diseases. Donors with any history of illness (e.g. all known infectious diseases, morbid obesity, diabetes, IBD, irritable bowel disease, chronic diarrhea, constipation, colorectal polyps, cancer, immunocompromised states, metabolic syndrome, allergy, chronic fatigue syndrome, genetic disease and major gastrointestinal surgery) were excluded. Other diseases or conditions potentially associated with specific changes in gut microbiota were also excluded. All the donors were assessed by laboratory evaluation, including complete blood count, C-reaction protein (CRP), erythrocyte sedimentation rate (ESR), IgM, IgA, IgG and biochemical test. Chronic hepatitis B and C, syphilis, and human immunodeficiency virus were excluded to avoid the transmission of these diseases. Donor stools were tested for C difficile, ova and parasites. Importantly, donors’ family health history, personal psychological health, personal credibility (potential cheating) and living environment (typical heat, cold, dry, or polluted industrial area) were assessed. Donors had to meet all the selection criteria.

### Study design

**Figure 4:**
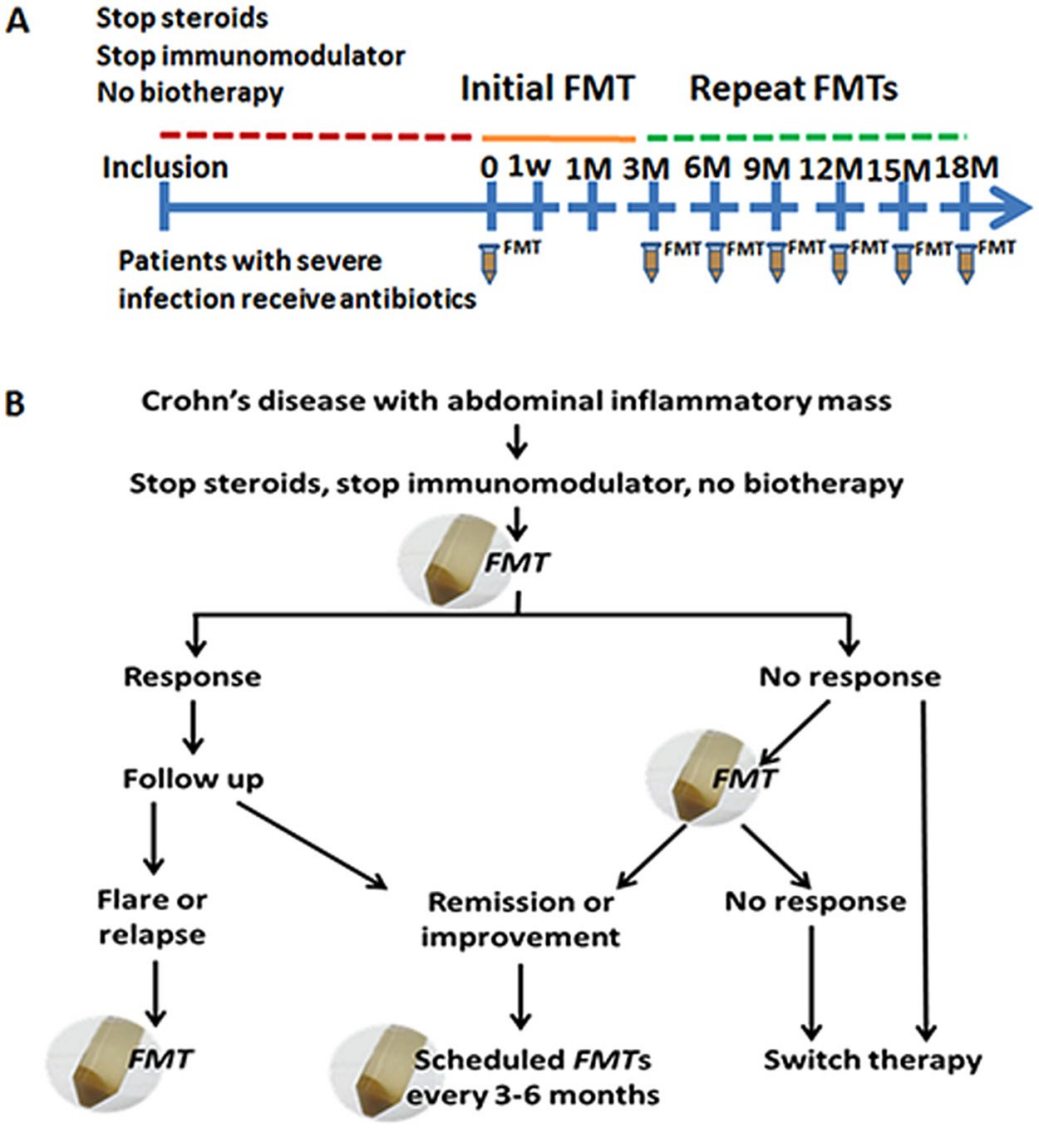
Study Design (**A**) and Multiple FMTs schedule (**B**).

The study design is shown in Fig. 4A. Baseline data collected from CD patients included demographic information, disease duration and location, Harvey-Bradshaw Index (HBI) and the characteristics of their intraabdominal inflammatory masses. The steroid, antibiotics and other CD medications were required to be stopped after inclusion. Patients with severe bacterial infections according to blood test were allowed to continue antibiotics until the time of their initial FMT. Mesalazine 3.0 g daily was given as a supplemental treatment for three months and then at a reduced dose of 1.5–2.5 g daily according to our protocol^12^. Parenteral nutrition plus partial enteral nutrition were administered to support those patients with severe malnutrition or intestinal stricture during hospitalization. The timeframe from enrollment to the initial FMT was not unified but generally within one week, because they were under refractory situation. Patients received the initial fresh FMT and subsequent multiple fresh FMTs every 3 months (Fig. 4A). Clinical assessments were performed at one week, one month, three months and then every three months after the initial FMT. All patients were followed up for a minimum of 12 months after the initial FMT. The primary endpoint was clinical response (improvement and remission) and sustained clinical remission at 12 months. Secondary endpoints were improvement in size of phegmon/abscess based upon cross-sectional imaging and safety of FMT. The laboratory tests at each visit were also evaluated, which included complete blood count, CRP, ESR and Hemoglobin. The following events were regarded as relapse or failure: (1) required urgent surgical procedures, (2) switched to other therapies, (3) did not respond clinically or worsened.

### Multiple FMTs

The study schedule for multiple FMTs is shown in Fig. 4B. Patients who showed response to the initial FMT were scheduled to receive subsequent FMTs every 3 months as maintenance. Patients who failed to benefit from the initial FMT were suggested to undergo a second FMT within one week^14^. The inflammatory makers and HBI score were used to assess for possible Crohn’s flare or potential relapse. A relapse at any time was managed with FMT to induce remission or improvement. The patients who failed to benefit from any FMT could switch to other therapy, including but not limited to antibiotics, surgery and biologic therapy.

### Preparation of fecal microbiota and FMT

Our original FMT preparation method, termed Filtration plus Centrifugation (FPC) and subsequently a newly developed automatic purification system named GenFMTer (FMT Medical, Nanjing, China) were used to purify the fresh stool^12–14^. Our FMT protocol was designed to be completed within one hour (also called as ﻿the “one-hour FMT protocol”), from ﻿stool fresh collection to transplantation or storage, using the support of the automatic system GenFMTer^13^. In short, the general procedure includes filtration, centrifugation, washing, discarding and dilution. Since 2014, stool collection and all experiments on stool were performed in a Good Manufacturing Practice level laboratory (see Supplementary Figure 1)^13^. The fresh stool was collected in a disposable bucket, which was designed for the GenFMTer machine. For 23 of the 25 study patients, the prepared microbiota liquid suspension was transplanted into the distal duodenum of patients through gastroscope under anesthesia. In order to prevent the microbiota liquid reflux and inhibit gastric acid secretion, patients were given metoclopramide 10 mg by intramuscular injection and esomeprazole magnesium 40 mg intravenously one hour before FMT^12^. Two of the 25 patents underwent a novel way of transplanting fecal microbiota using transendoscopic enteral tubing (TET)^24^, which consists of placing a tube through the endoscope through the anus into the cecum for whole colon administration of fresh fecal microbiota suspensions. The psychological protection and caring for patients undergoing FMT was one of important clinical work-flow quality managements.

### Efficacy and safety assessment

Patients underwent clinical evaluation at each visit with measurement of HBI as well as laboratory tests as described above. Clinical remission was defined as HBI score ≤ 4. Sustained clinical remission was defined as remission induced by the initial FMT within one month and then maintained remission (without any relapse) under FMTs before each assessment point during the follow-up. Clinical improvement was defined as a decrease of HBI > 3. A relapse was defined as symptoms and signs of recurrence and HBI score >4, or a disease flare requiring use of infliximab or surgery. Clinical response included clinical improvement and clinical remission. Patients with no significant clinical improvement were deemed non-responders. In the analysis for the rate of clinical remission or response, the patients who did not reach the required assessment point were not involved. All patients underwent MRI scanning according to our protocol^25^. Patients who had contraindication to MRI were scanned by CT. All enrolled patients with imaging review received the evaluation of intraabdominal inflammatory mass and classified as healed, improved, unchanged, or worse. Each MRI or CT was assessed in consensus by two experienced gastrointestinal radiologists who were blinded to the clinical outcome. The subsequent MRI or CT scan was compared with the baseline scan and the previous scan. The size of inflammatory mass was the maximum diameter times the vertical short diameter in the largest cross section. Radiological healing was defined as complete resolution of inflammatory mass. Radiological improvement was defined as estimated reduction of 10% or more in the size of the inflammatory mass. No radiological change was defined as any reduction less than 10% in the size of the inflammatory mass. Worsening of the radiological was defined as an increase of at least 10% in the size of the inflammatory mass. Adverse events were recorded during FMT and throughout the follow-up to evaluate the safety. Common Terminology Criteria for Adverse Events (version 3.0) was used to describe the intensity and relationship of adverse events with FMT. Intensity of adverse events was classified as mild, moderate, severe, or disabling. Relationship of adverse events with FMT was categorized as unrelated, possible, probable, or definitely related to FMT.

### Statistical analysis

Data were analyzed using SPSS 15.0 (Chicago, IL, USA). Data that conforms to the normal distribution was expressed as mean ± standard deviation. Data that does not conform to the normal distribution was expressed as median (Interquartile range, IQR) When the normality of the distribution of variables was acceptable, the paired student’s t test was used. When the normality of the distribution of variables was not acceptable, the Wilcoxon signed-rank test was used to analyze differences between groups. P values < 0.05 were considered significant.

### Ethical Considerations

This pragmatic prospective experimental study was registered at Clinicaltrials.gov (NCT01793831) on 13 February 2013 and was carried out by the Medical Center for Digestive Diseases at the Second Affiliated Hospital of Nanjing Medical University, China, from October, 2012 to September, 2016. The study protocol was approved by Medical Research Ethics Committee of the Second Affiliated Hospital of Nanjing Medical University. All participants provided written informed consent. We confirmed that all methods were performed in accordance with the approved guidelines and regulations. We reported and presented data according to the CONSORT statement.

## Electronic supplementary material

Good Manufacturing Practice level laboratory for FMT
