# Supplementary material for: Multiple fresh fecal microbiota transplants induces and maintains clinical remission in Crohn’s disease complicated with inflammatory mass
Source: Sci Rep. 2017 Jul 6;7:4753. doi: 10.1038/s41598-017-04984-z (PMC5500501; doi:10.1038/s41598-017-04984-z)

Multiple fresh fecal microbiota transplants induces and maintains clinical remission in Crohn's disease complicated with inflammatory mass

Zhi He[1,2], Pan Li[1,2], Jianguo Zhu[3], Bota Cui[1,2], Lijuan Xu[1,2], Jie Xiang[1,2], Ting Zhang[1,2], Chuyan Long[1,2], Guangming Huang[1,2], Guozhong Ji[1,2], Yongzhan Nie[4], Kaichun Wu[4], Daiming Fan[4], Faming Zhang[1,2]

Supplementary Information

Good Manufacturing Practice level laboratory for FMT

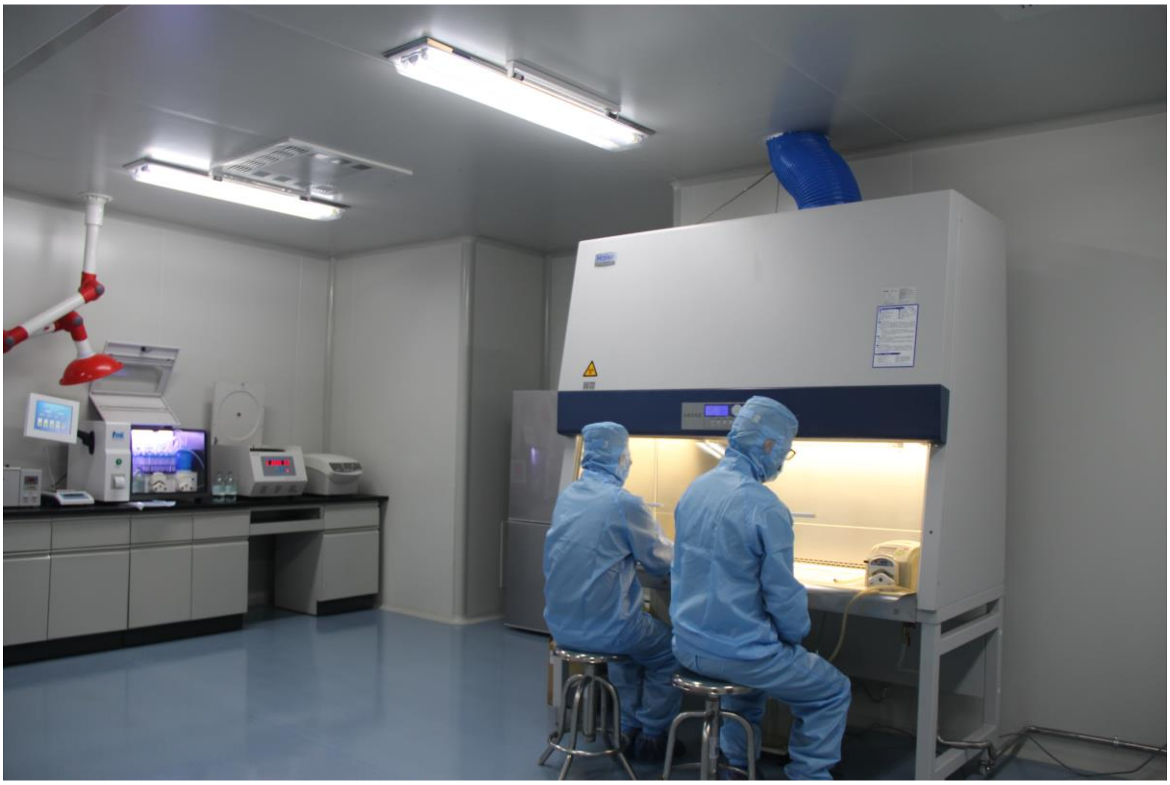

Supplement: Supplementary file 1 — Good Manufacturing Practice level laboratory for FMT [file 41598_2017_4984_MOESM1_ESM.pdf]
